# ‘The Pandemic Will Not be on Zoom’: A Retrospective from the Year 2050

**DOI:** 10.1007/s42438-020-00150-3

**Published:** 2020-07-03

**Authors:** Eamon Costello, Mark Brown, Enda Donlon, Prajakta Girme

**Affiliations:** 1 National Institute for Digital Learning, Dublin City University, Dublin, Ireland; 2 Institute of Education, Dublin City University, Dublin, Ireland

**Keywords:** Covid-19, Decolonizing the curriculum, Post-truth, Speculative fiction

## Introduction

**Fig. 1:**
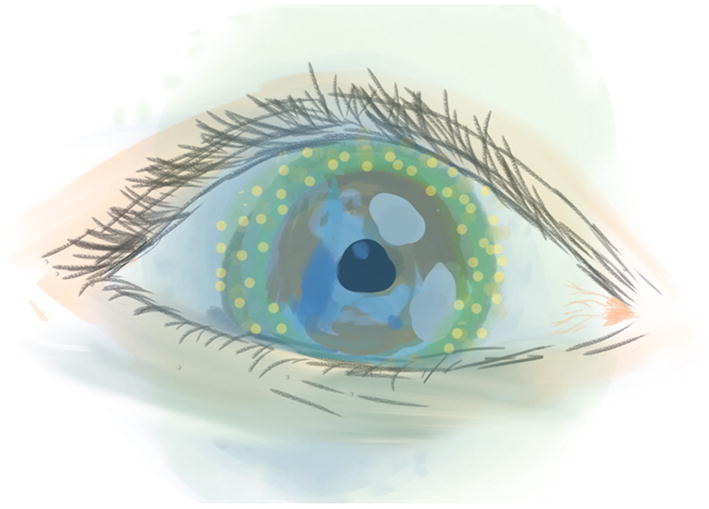
Depiction of the deepmind hive AI dated circa 2034

This paper aims to interpret, analyse, and critique educational pasts, presents, and futures. It is framed by potentially falsifiable memories of colonization and struggles for identity and social justice. We adopt the device of social science fiction (Gerlach and Hamilton 2003) as a specialist genre of speculative fiction (Graham et al. 2019). Such speculative approaches seek to develop provocations rather than predictions (Selwyn et al. 2020) and to implicate their readers rather than to inculcate them. In this tradition, we seek to ponder possibilities of post-pandemic educational futurities. Our work centres on the ramblings of an unknown scholar who, on the cusp of a post-scientific world, screams a maddened poem into the void titled ‘The Pandemic will not be on Zoom’. The events surrounding this poem are pieced together to reveal a world of stark inequities and digital and biological fractures. These fractures prefigured a bleak colonization of humankind by a deepmind hive Artificial Intelligence (AI) (Fig. 1) that caused us to become forever isolated from ourselves and that brought an end to the grand projects of science and education. In our conclusion, we call for other historians of futures past to help uncover timelines, and write alternative fictions, that promote pedagogies of hope, care, justice, and a brighter day.

## To 2050

**Fig. 2:**
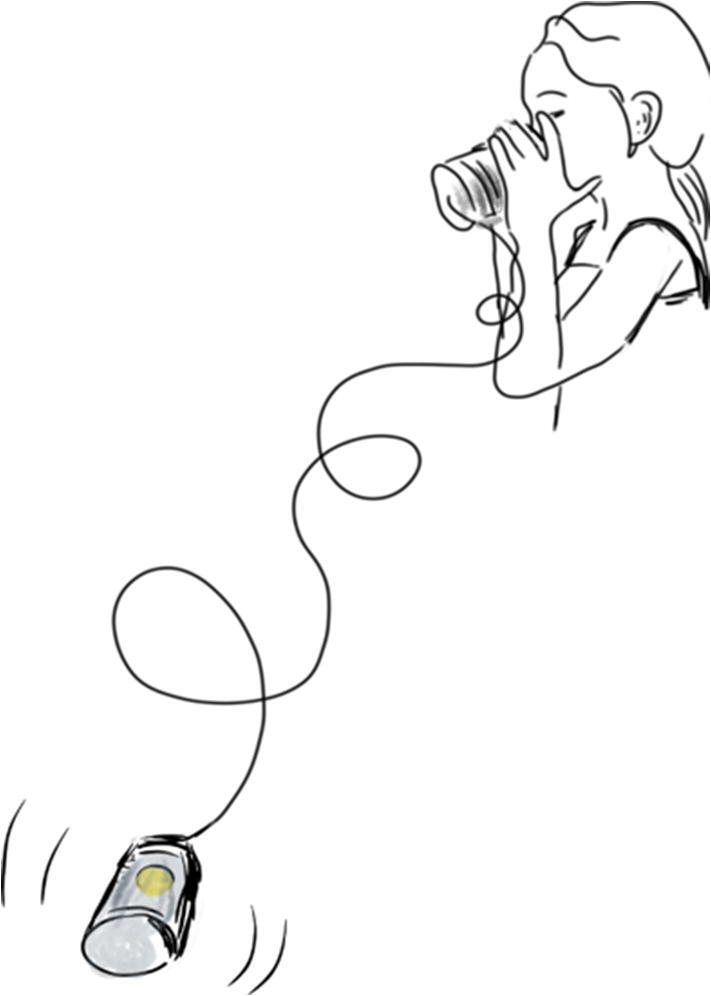
Humanity isolated from itself

The year is 2050. An insatiable human instinct to consume the flesh of fellow animals had industrialized and mechanized agriculture, penning countless livestock wing-to-wing, hoof-to-hoof, and snout-to-snout in a vast sprawl of cramped enclosures. Outside of these factory farms, an increasingly scarce yet diverse population of wild animals were hunted, trapped, and brought to market for human consumption. In this cauldron, people, animals from earth’s crevices, and mysterious microbes were stewed in ever more novel ways over centuries of ingenious and increasingly intensive dominion and pillage by humankind of their natural world. A deadly wave of pathogens was precipitated. Pandemics swept the earth, periodically over several decades. As they raged, the nature of human life on earth was radically reconstituted. The Age of the Great Distancing was born. This era came about by a simple, cunning characteristic of the diseases. The pathogens had evolved to ride upon a particular transmission vector. They had learned our human impulse for closeness, for touch, for affection, for compassion. They travelled on the bodily fluids that we expelled and exchanged in the expressions of what we believed were core to our purpose and being. They preyed on love and all of its small antecedents and shy proxies. They fastened themselves to everything that made us human. They learned that the human body was ‘a new form of interface’ (Facer 2011: 63). Thus they spread, faster than the ability of all of the then available science and politics to control. Humanity was remade—now forever isolated from itself (Fig. 2).

A concomitant, non-physical revolution began. Fired by advances in AI, building on the ideas of the late Edmond Kirsch, a billionaire philanthropist, the Internet undertook a step change in complexity, connectedness, and power. As if responding to humans’ need for touch, the emerging deepmind hive began to reach up to comfort us. We fell willingly into its digital embrace, feeling its cold fingers upon our backs. We groped its digital flesh, safe from the biological predations of the enemy. In its digital corporeal promise of uncomplicated connectedness we felt safe—isolated, disconnected from fears and emotions, cocooned from our deceptive and deadly physical truths.

## Making History

Insights into the changing world were continually provided by deepmind hive AI historians, but it is also worth noting that pre-posthuman, fully wet bodied, historians had also written passably on these matters. These early wet-ware historians used time travel to scrutinize various aspects of history. Education was a particular focus of their examinations. For example, by 2040, the historians Macgilchrist et al. (2020) had detected three alternative timelines of education’s history that described alternately:

‘smooth users’, improving themselves in the pursuit of frictionless efficiency within a post-democratic frame created by large corporations [;] ‘digital nomads’, seeking freedom, individualism and aesthetic joy as solopreneurs exploiting state regulations and algorithmic rules while stepping out of the state and deeply into the capitalist new economy [; and] participatory, democratic, ecological humans embedded in ‘collective agency’ that see institutions as spaces for exploring more equitable ways of living. (Macgilchrist et al. 2020)

Further back, in 2030, historians had posited electricity as a prototypical artefact of educational formulations: an equivalent to textbooks, lecterns, mortarboards, credit hours, lessons, and so on but one liable to be siphoned off to fuel alternative enterprises at the whim of the state (Selwyn et al. 2020). Interestingly, electricity became the backbone of ‘self-executing blockchain neighbourhoods’ (Zook 2019) as early as 2020, setting labourers to physically toil under encryption coloured skies, mining sexual consent contracts that draped themselves upon blockchains. This physical labour recalls the distinction between ‘virtual’ and ‘digital’ because the ‘digital requires bodies’, labouring bodies, as ‘the industrial production of digital technology delves beneath the surface of slick devices and their marketing campaigns to highlight the very real labour conditions through which they are assembled’ (Knox 2019: 366). These are the bodies Adebisi speaks of:

We bury the damned and wretched, for we expect the water to always hide them, but the stench of bloated bodies will rise from this wretched earth. We cannot decolonise the university using the same logics that made it a colonising force – the episteme that became a most effective and self-sustaining war machine. How illogical is it that the structure we are attempting to decolonise is the structure we are attempting to use to decolonise? (Adebisi 2019)

By 2050, historians had detected, and could start to describe, the darkest possible timeline: that of the Era of the Pandemics and the subsequent Age of the Great Distancing. Thus, they began to piece together its origins. The relevance for the history of education was quite striking. Although truth had been on extended life support (Fuller and Jandrić 2019) since a vicious attack by French philosophers in the latter half of the twentieth century—see Farrow and Moe (2019) for an overview—it finally evaporated during the Era of the Pandemics. Once a simple pathogen could defy every description, prediction, or claim of science, truth was finally abandoned as worthless.

**Fig. 3:**
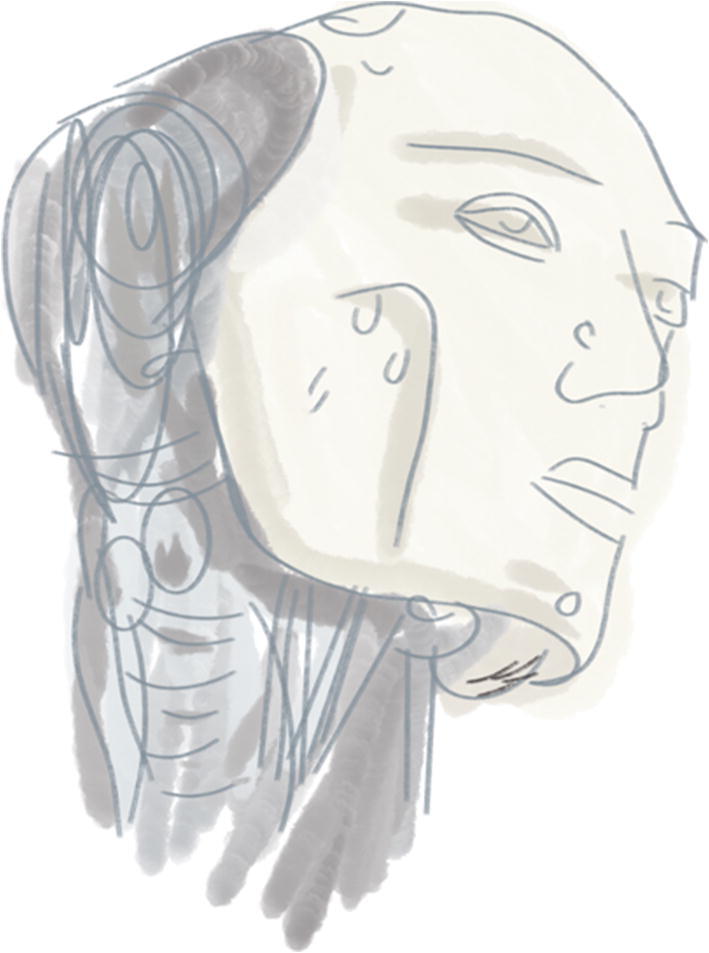
A ‘datafied’ student as depicted by a former academic

The first casualty of this bleak post-truth epoch was of course scientists and academics. Published research was deemed taboo, and engaging in scholarly activity was outlawed. This change was perhaps best exemplified by strange new forms that arose as replacements of scholarly writing. The first of these forms were sketches made by former scholars depicting abstract fragments of their new reality. We include curious examples from one academic artist in this article (see Figs. 1, 2, and 3). Their exact nature and provenance is unknown though historians have dubbed the artist ‘Lily’. The second replacement of scholarly activity was the rise of academic poetry and rap. One notable early dirge will help to exemplify this trend. Written by an unknown scholar, it comprised a grim pastiche, or incorporation, of an olden song that drew on issues of race relations and their media portrayal (Gill Scott-Heron 1970). This co-option, by the anonymous scholar, was the final indication that the colonization of academia had been completed, as the colonizer was now the colonized (Swift 1792; Costello 2020). An extract from this piece is reproduced below as an illustrative example of the rapid deterioration of the mental health of scholars. The writer is clearly showing early onset of a psychosis evidenced by an inability to discern, or bound, the realities of his or her personal and professional identities:

## The Pandemic Will Not be on Zoom

The Pandemic will not teach you algebra when old people equal zero

The Pandemic will not prepare you for jobs that do not exist yet when jobs do not exist

The Pandemic will not give you tenure

The Pandemic will not be graded

The Pandemic will not be sponsored by TurnThemIn, RetinaScanMyEssay or X-RayedLearner

The Pandemic will not cite your work

The Pandemic will not be patented, copyrighted, contained or explained

The Pandemic will not be described in 500 words by next Friday because the Pandemic will not be on Zoom

The Pandemic will not have an ISBN, a DOI, a URL or a page number while children know the words, PPE, RNA, Attack Rate and ICU

The Pandemic will not come with instagrammed brie on rosemary ciabatta drizzled with balsamic vinegar when factory farms birth the next pathogen

The Pandemic will not social distance when animals live in cages

The Pandemic will not have a Gap Year

The Pandemic will not give you a beach body for Spring Break when army trucks collect coffins from churches in Bergamo

The Pandemic will not make you go viral, increase your reach, amplify your message or make you look good

The Pandemic will have no alumni

The Pandemic will not release you from gaol because the PATTERN algorithm sings that white collar crime is white crime is right crime

The Pandemic will not have new book smell when students sell plasma to go to College

The Pandemic will not be recorded so you can watch back later

The Pandemic will not be recorded so you can watch back later because the Pandemic will not be on Zoom

The Pandemic will not make you a well-rounded citizen

The Pandemic will not ace the test when Dr. Lorna Breen recovers from infection goes back to work and then takes her own life

The Pandemic will not have a diversity officer a lesson on probability, or a history class when people of colour die in huge numbers

The Pandemic will not have an Acknowledgements Section

The Pandemic will not sell merch

The Pandemic will not have a Call for Papers a Special Issue, an APC or a Grant Number

The Pandemic will not be world class when Li Ren says goodbye to her family to stay with an orphaned four-year-old in a quarantine centre and chooses not to wear full PPE so the girl can see more of her face

The Pandemic will not have a deadline

The Pandemic will not have a parent teacher meeting because parents are too busy to meet themselves

The Pandemic will not collect, store, safeguard, sell, steal, smell, taste or breathe your data in any way whatsoever because the Pandemic will not be on Zoom.

Historians have since identified several of the events outlined in the crude and badly written verse. It was confirmed that an algorithm designed to predict recidivism and release prisoners during the pandemic, contained positive biases towards white people and ‘white collar crime’ (Hager 2020). There were contemporaneous corollaries in education that the unstable scholar would have been familiar with, such as the ‘datafication’ of students. Datafied students could be subject to algorithms that held sway over them to such a degree that educators could worry that ‘prediction of future progress based on past outcomes could radically affect the future prospects of the student by foreclosing curriculum opportunities’ (Williamson et al. 2020). Figure 3 below shows an example of how a scholar of the time believed a datafied student might manifest.

Historians were also surprised to learn about textbook poverty, about the inflationary costs of textbooks that scholars had vainly struggled to resist (Hilton 2019; Clinton and Khan 2019; Costello et al. 2018; Weller et al. 2015). Historians have confirmed that medical companies did indeed target students, offering to buy their blood plasma, so that the students could buy books (Sander 2019).

By 2049, the deepmind hive AI historians had detected the fatal flaws that had finally collapsed knowledge production. Even scholars of distant eras had dim ideas that citations were not really correlates of truth but instead a form of virtue signalling and primitive gift giving. What was less understood at the time, although it had been foreshadowed (Kuhn 2012), was that science was really a giant socially constructed illusion, ‘a spume that plays upon a ghostly paradigm of things’ (Yeats 1928).

## Prefiguration

There is a device of the telling, dear reader that must be briefly explicated at this juncture, before humanity undergoes its ultimate colonization. The literary trope of relevance is prefiguration. Or what might be also termed here—a sensitization to sacrifice. Prefiguration seeks to normalize the calculus of who can be killed so that others may live (Mbembé and Meintjes 2003; Adebisi 2019). It was thus that a blind scribe, in foretelling a lost paradise, used a surgical wound in an opening verse to prefigure the blood sacrifice of Christ (Dobranski 2015). It is through the death match in the ‘kill-zone’ that empires are conceived and from which colonisations can propagate (Tuck and Yang 2012). So too our tale now needs a sacrificial prophet.

Historians have highlighted the curiosity of ‘Open Science’, a strange tragi-comedic form of the early twenty-first century that appeared to imply the existence of truth. This truth, it was inferred, could be attained via quixotic quests composed of specific shamanic ‘open’ scientific rituals. One prominent scientist of this cult published a paper titled ‘Why most research findings are false’ (Ioannidis 2005). The paper, attempting to herald a reboot of truth, garnered several thousand citations and millions of downloads. (With hindsight, these occult portends in and of themselves might have been heeded.) During the first pandemic, Ioannidis was charged with the sin of engaging in the very same poor scientific practices he had built a career attacking (Schulson 2020). As his followers turned on him, the final sacrifice was enacted—the hyper-reality (Baudrillard 1988) of science could reveal itself as a self-propagating fiction constantly devouring itself in a shimmering ouroborus. The future could now unfold itself as a story in which every word was the same, ‘a language on a monoplain’ (Enan 2009a).

As the bodies mounted, and science fell into an irreparable disrepute, the problem became clear. It was our humanity: we were ‘human, all too human’ (Nietzsche 1878, 1908). Thus, we hastened our embrace of the deepmind hive and our post-humanity. Teachers and scientists were finally fully learnified away (Biesta 2012). Education, which had long vied with prostitution for the title of the world’s oldest profession, was irrevocably ended.

## Treading Other Timelines

There are of course other potential timelines. We invite other historians of past futures (Fuller and Jandrić 2019) to help uncover these through their own fictions. Perhaps there are brighter post-human histories, which shine light on different paths, avoiding the deadly pedagogies of the depressed. Ones where we ‘coalesce into physical relationships and groupings that belong to a higher order’ (de Chardin 2012: 153) where we find ‘the life more than the food, and the body than the raiment’ (Matthew 6:25). Perhaps, a timeline will show how education provided a vital sense-making activity for people. Perhaps, it will show how they came together in the practice of this activity in a time of deep turmoil. Perhaps, we ditched biodiversity for nature. Perchance, we felt our animal skins, ‘the sand through our feet’ (Enan 2009b)—turned our backs on electricity’s dark fictions (Alexander 2020) and gathered around the warm flames of a campfire, in a clearing, deep within ‘the jungle of justice’ (Watters 2020). Perhaps, we learned to see the people at ‘the edge, the periphery, the margins of our human scatter-plot [...] the outliers and weak signals’ (Treviranus 2019). Perhaps, we paved the way for new postdigital pedagogical ecologies (Bayne 2018) of justice and care (Bali and Sharma 2014; Cronin 2020) where we could peacefully ‘inter-be’ (Hanh 2010). Perhaps, it opened the door to a brighter day (Scott-Heron 1970) where people could once again step outside, touch each other, and be okay.
